# Novel neutralizing SARS-CoV-2-specific mAbs offer detection of RBD linear epitopes

**DOI:** 10.1186/s12985-024-02304-2

**Published:** 2024-02-06

**Authors:** Seyed Mostafa Mostafavi Zadeh, Ali Ahmad Bayat, Hosein Shahsavarani, Feridoun Karimi-Busheri, Jafar Kiani, Roya Ghods, Zahra Madjd

**Affiliations:** 1 Oncopathology Research Center, Iran University of Medical Sciences, Tehran, Iran; 2 Department of Molecular Medicine, Faculty of Advanced Technologies in Medicine, Iran University of Medical Sciences, Tehran, Iran; 3 Monoclonal Antibody Research Center, Avicenna Research Institute, ACECR, Tehran, Iran; 4 Laboratory of Regenerative Medicine and Biomedical Innovations, Pasteur Institute of Iran, National Cell Bank, Tehran, Iran; 5 Department of Cell and Molecular Biology, Faculty of Life Sciences and Biotechnology, Shahid Beheshti University, Tehran, Iran; 6 Department of Oncology, Faculty of Medicine, University of Alberta, Edmonton, AB T6G 1Z2 Canada

**Keywords:** Linear epitope, Monoclonal antibody, Neutralizing antibody, RBD, SARS-CoV-2

## Abstract

**Background:**

To stop the spread of the COVID-19 disease, it is crucial to create molecular tools to investigate and diagnose COVID-19. Current efforts focus on developing specific neutralizing monoclonal antibodies (NmAbs) elicited against the receptor-binding domain (RBD).

**Methods:**

In the present study, recombinant RBD (rRBD) protein was produced in E. coli, followed by immunizing mice with purified rRBD. ELISA was applied to screen the hybridomas for positive reactivity with rRBD protein. The linear and conformational epitopes of the mAbs were subsequently identified using western blot. Finally, the reactivity, affinity, and neutralization activity of the purified mAbs were evaluated using ELISA.

**Results:**

All mAbs exhibited similar reactivity trends towards both eukaryotic RBD and prokaryotic rRBD in ELISA. Among them, 2E7-D2 and 2B4-G8 mAbs demonstrated higher reactivity than other mAbs. Additionally, in western blot assays, these two mAbs could detect reducing and non-reducing rRBD, indicating recognition of linear epitopes. Notably, five mAbs effectively blocked rRBD- angiotensin-converting enzyme 2 (ACE2) interaction, while two high-affinity mAbs exhibited potent neutralizing activity against eukaryotic RBD.

**Conclusion:**

In the current study, we generated and characterized new RBD-specific mAbs using the hybridoma technique that recognized linear and conformational epitopes in RBD with neutralization potency. Our mAbs are novel candidates for diagnosing and treating SARS-CoV-2.

**Supplementary Information:**

The online version contains supplementary material available at 10.1186/s12985-024-02304-2.

## Background

To stop the spread of the COVID-19 disease, several attempts are being made to create efficient medications and develop novel treatment strategies [1]. No specific cures can entirely treat the disease caused by severe acute respiratory syndrome coronavirus 2 (SARS-CoV-2) [2]. Thus, developing effective and safe therapeutic agents is urgently needed. Fortunately, antiviral therapies, including immune globulins and monoclonal antibodies, can precisely and efficiently recognize targets, while they have few side effects in humans [3]. Major research has focused on identifying antiviral compounds that target and inhibit the activity of S proteins, which potentially play a significant function in virus entry in the host cell [4]. Preclinical/clinical studies have indicated that anti-SARS-CoV-2/RBD has a critical role in the adaptive immune response, one of the most significant roles of protection in infectious diseases [5, 6]. In addition to producing preventive vaccines, the passive administration of monoclonal antibodies (mAbs) may be the key to controlling the SARS-CoV-2 pandemic by offering immediate protection [7]. Thus, neutralizing monoclonal antibodies (NmAbs) against SARS-CoV-2 has become a promising strategy by blocking viral entry into target cells [5, 8] that can reduce viral burden by preventing viral spread after infection [9]. Furthermore, NmAbs neutralize viral infection or replication by targeting viral proteins, including the spike (S) glycoprotein, and facilitate the clearance of viruses via Fc-mediated effector functions [10, 11].

Based on the evidence, most of the SARS-CoV-2 neutralizing antibodies (nAbs) are directed against the S1 subunit of the S protein [12]. The S1 subunit has two major structural domains, receptor-binding domain (RBD) and N-terminal domain (NTD) that interact with the angiotensin-converting enzyme 2 (ACE2) receptor and nAbs are especially against the RBD [13]. Some of these antibodies have been described with therapeutic or prophylactic functionality against SARS-CoV-2 in animal models [13].

Considering the impact of SARS-COV-2 pandemic on global health, there is an immediate requirement to develop potent NmAbs that can effectively neutralize the virus to manage infection and disease progression. The study focuses on producing a recombinant RBD (rRBD) protein in E. coli BL21(DE3) and generating of NmAbs targeting the rRBD of SARS-CoV-2 using hybridoma technology. Subsequently, these NmAbs were analyzed to evaluate their potential for passive immunotherapy use.

## Materials and methods

### Preparation of immunogen

#### Construction of vector and rRBD expression

The pET22b expression vector (Novartis, USA), encoding residues 319–541 of the SARS-CoV-2 S protein sequence from strain delta (GenBank ID: YP_009724390.1), was transformed to E. coli expression strain BL21(DE3). Freshly transformed E. coli were grown in LB broth in a shaker (200 rpm) at 37 °C in a total volume of 50 ml that contained 100 µg/ml of ampicillin until the OD_600_ value reached 0.8–1.0 (about 4–5 h). Isopropyl-β- D-Thio-Galactopyranoside (IPTG) was added to the final concentration of 0.1 mM, and then bacteria were induced 3 h at 37 °C. After induction, the bacteria were harvested by centrifugation at 8,000 g for 15 min at 4 °C. The pellet was washed with 200 ml of 50 mM Tris–HCl buffer (pH 8.0) containing 5 mM EDTA and 1 mM PMSF and centrifuged again at 18,400 g for 15 min at 4 °C. The inclusion bodies were washed with 50 mM Tris–HCl buffer (pH 8.0) containing 5 mM EDTA and 2% deoxycholate. The inclusion bodies were again resuspended and solubilized in 5 ml lysis buffer (100 mM Tris–HCl, 100 mM NaH_2_PO_4,_ and 8 M urea, pH 8.0) and sonicated on ice 15 × 20 s with a 50% duty cycle at 75% power [14]. After high-speed centrifugation, bacteria pellets and supernatant samples were placed on 12% SDS-PAGE gels, and protein bands were visualized by Coomassie Brilliant Blue staining.

#### Purification of SARS-CoV-2 rRBD protein

After the solubilization of inclusion bodies, the Ni–NTA chromatography column (Noavaran Zistgostar, Iran) was pre-equilibrated with lysis buffer solution (pH 8.0). The RBD supernatant was applied to the column. After sample loading, the column was washed with 5 ml of washing buffer (100 mM Tris–HCl, 100 mM NaH_2_PO_4_, and 8 M urea, pH: 6.3). Following that, the rRBD protein was eluted with elution buffer (100 mM Tris–HCl, 100 mM NaH_2_PO_4_ and 8 M urea, pH 4.5).

The concentration of purified protein was calculated by the bicinchoninic acid (BCA) assay [15]. Then, the purity and identity of the antigen were assessed using the SDS-PAGE and western blot, respectively.

#### SDS-PAGE and western blot

All protein fractions and purified rRBD protein was subjected to SDS-PAGE electrophoresis (12%), and the protein was stained by Coomassie blue dye R250 (Sigma Aldrich, Germany). Another sample was separated by non-reducing and reducing SDS-PAGE and transferred to polyvinylidene difluoride (PVDF) membranes (Roche, Mannheim, Germany) using an electrophoresis system (Bio-Rad). Membrane blocking was performed with 5% skim milk overnight at 4 °C. After washing with phosphate-buffered saline (PBS)/Tween-20 buffer, the membrane was incubated with HRP-anti-His tag (BioLegend, USA) (1:10,000) for 1.5 h at 37 °C. After washing, the rRBD protein was visualized by 3,3′-Diaminobenzidine (DAB) (Sigma, USA) color development solution. For evaluation with human serum, the membrane was incubated with positive human serum (SARS-CoV-2 immunized serum) and negative serum (collected before the COVID-19 pandemic) at dilutions of 1/1000 for 1.5 h at 37 °C. After washing, membranes were incubated for 1.5 h with HRP-conjugated goat anti-human IgG (Avicenna Research Institute, Iran). The bands were visualized with enhanced chemiluminescence (ECL) (Thermo Fisher Scientific™, USA).

### Immunization procedure

The rRBD protein was prepared in an appropriate volume of PBS (100 μl) and mixed with Freund's adjuvant at a ratio of 1:1 (v/v). The first injection of 2 female Balb/c mice (4 to 6 weeks old) was performed with an emulsion containing an equal volume of RBD solution and Complete Freund’s adjuvant mixture (50 μg of antigen per dose) via intraperitoneal (IP) route. Immunization was performed every 2-week intervals (3 times), containing 25 μg of antigen emulsified in Incomplete Freund’s adjuvant. After 8 weeks of the first immunization, blood was collected to assess the RBD-specific mouse IgG titer by ELISA. The hyperimmunized mice were selected for mAb production. Final immunization was performed with 25 μg of antigen via the tail vein [16, 17].

### Splenectomy and fusion

The immunized mice were anesthetized, and the spleen was aseptically removed. Dispersed splenocytes and sp2/0 cells were mixed at a ratio of 5:1, followed by washing 3 times with 10 ml serum-free medium (RPMI, Gibco, USA) at 400 × g for 5 min, then 800 μl of PEG (Polyethylene glycol solution) (Merck, Germany) added dropwise to cells. The cell mixture was dissolved in RPMI and centrifuged 3 times, 275 g at 37 °C for 5 min [16].

### Hybridoma cloning and selection

HAT 1X Medium (hypoxanthine-aminopterin-thymidine medium) (Sigma, USA) containing FBS 10 ml, non-essential amino acid 0.5 ml, pyruvate sodium 0.5 ml, HAT 50X 2 ml, and RPMI medium 37 ml was used for hybridoma selection. Briefly, mixture cells (splenocytes and sp2/0 cells) were harvested and suspended (4.3 × 10^7^ cells) in a 20 ml HAT medium, plated (200 μl/well) into a 96-well cell culture plate (SPL Life Sciences-Korea), and incubated at 37 °C in a 5% CO_2_ humidified incubator for 7–10 days. After 2 and 4 days, 100 μl of the medium was changed with fresh HAT medium. Indirect ELISA was performed for the screening of cell supernatants. Hybridoma with specific antibody production was cloned five times by limiting dilution [17, 18].

### Purification of anti-rRBD mAbs

According to the manufacturer's instructions, mAbs were purified by a HiTrap protein G HP affinity column (GE Healthcare, Uppsala, Sweden). Then, antibody concentrations were calculated using an extinction coefficient of mouse IgG in 280 nm and purity of the mAbs was assessed by SDS-PAGE (10%). Mouse IgG (Avicenna Research Institute, Iran, MW: 150 Kd) was referenced in all mAbs purity assessments. Purified mAbs were aliquoted and kept at − 20 °C for storage.

### Indirect ELISA assay

ELISA was carried out for titration of mouse sera, screening of hybridomas, and reactivity assessment of purified mAbs. Briefly, the ELISA plate (SPL Life Sciences, Korea) was coated with rRBD (10 μg/ml in PBS) and then incubated at 37 ℃ for 1 h, followed by overnight incubation at 4 ℃. After washing, plates were blocked with bovine serum albumin (BSA) 2% for 1.30 h at 37 ℃. Subsequently, serial dilutions of immunized mouse sera or supernatants of hybridoma cells or purified antibodies were added, and plates were incubated at 37 °C for 1 h. Then, horseradish peroxidase-labeled (HRP) goat anti-mouse IgG (Hura Teb Pharmed, Iran) was added, and the optical density (OD) of 3,3′,5,5′-Tetramethylbenzidine (TMB) (Kiazist Pishro, Iran) was measured at 450 nm using a Synergy multi-mode reader. To assess the functionality of the rRBD, ACE2-HRP was directly added to the coated plate, and the OD was measured. In addition, similar to the above steps, the antibody reactivity was evaluated with the commercial kit (Pishtaz Teb Zaman, Iran) coated with eukaryotic RBD.

### Affinity assay

The affinity constant (K_aff_) of produced mAbs was determined by indirect ELISA. Briefly, different concentrations of the rRBD (8, 4, 2, 1, 0.5, 0.25, 0.125, and 0.0625 μg/ml) were coated in 96-well ELISA plates. Appropriate concentrations of the anti-rRBD mAbs (8, 4, 2, 1, 0.5, 0.250, 0.125, 0.0625, 0.0312, 0.0156, 0.0078, and 0.0039 μg/ml) were prepared in PBS/T and 100 µl were dispensed into antigen coated wells, then incubated at 37 °C for 1.30 h. Hundred µl of HRP-Goat anti-mouse IgG diluted 1 in 10,000 in PBS/T were added, and the plates were incubated at 37 °C for 40 min. After washing and adding the TMB substrate for HRP, the enzyme reaction was stopped by adding 100 µl of 1 N H_2_SO_4_. Sigmoid curves were plotted using the OD values obtained for different concentrations of mAbs [19].

### Reactivity assessment of mAbs by western blot analysis

Five μg of the rRBD was electrophoresed in 12% SDS-PAGE gel and transferred to the PVDF membrane. Membrane blocking was performed with 5% skim milk overnight at 4 °C. After washing with PBS/T buffer, the membrane was incubated with mAbs at 200 ng/ml concentration for 1.5 h at 37 °C. After washing, membranes were incubated for 1.5 h with HRP-conjugated goat anti-mouse IgG (Hura Teb Pharmed, Iran). The bands were visualized with an ECL solution.

### Blocking ELISA for detecting neutralizing antibodies

Similar to the indirect ELISA section, blocking ELISA was performed. Briefly, serial dilutions of purified anti-rRBD-mAbs were added to rRBD-coated plates for 1 h at 37 °C. Only PBS was added to the wells in the coating step in negative reagent control wells. After washing, HRP-ACE2 was added to the plates for 30 min. In addition, to confirm neutralizing activity, all mAbs were evaluated by eukaryotic RBD. Subsequently, the neutralizing activity percentage was calculated based on this formula.

$$\mathrm{Neutralization activity percent}=100-\frac{OD \left( RBD+mAb+ACE2\_HRP\right)\times 100}{OD \left( RBD+ACE2\_HRP\right)}$$

## Results

### Purity assessment and the rRBD characterization

**Fig. 1:**
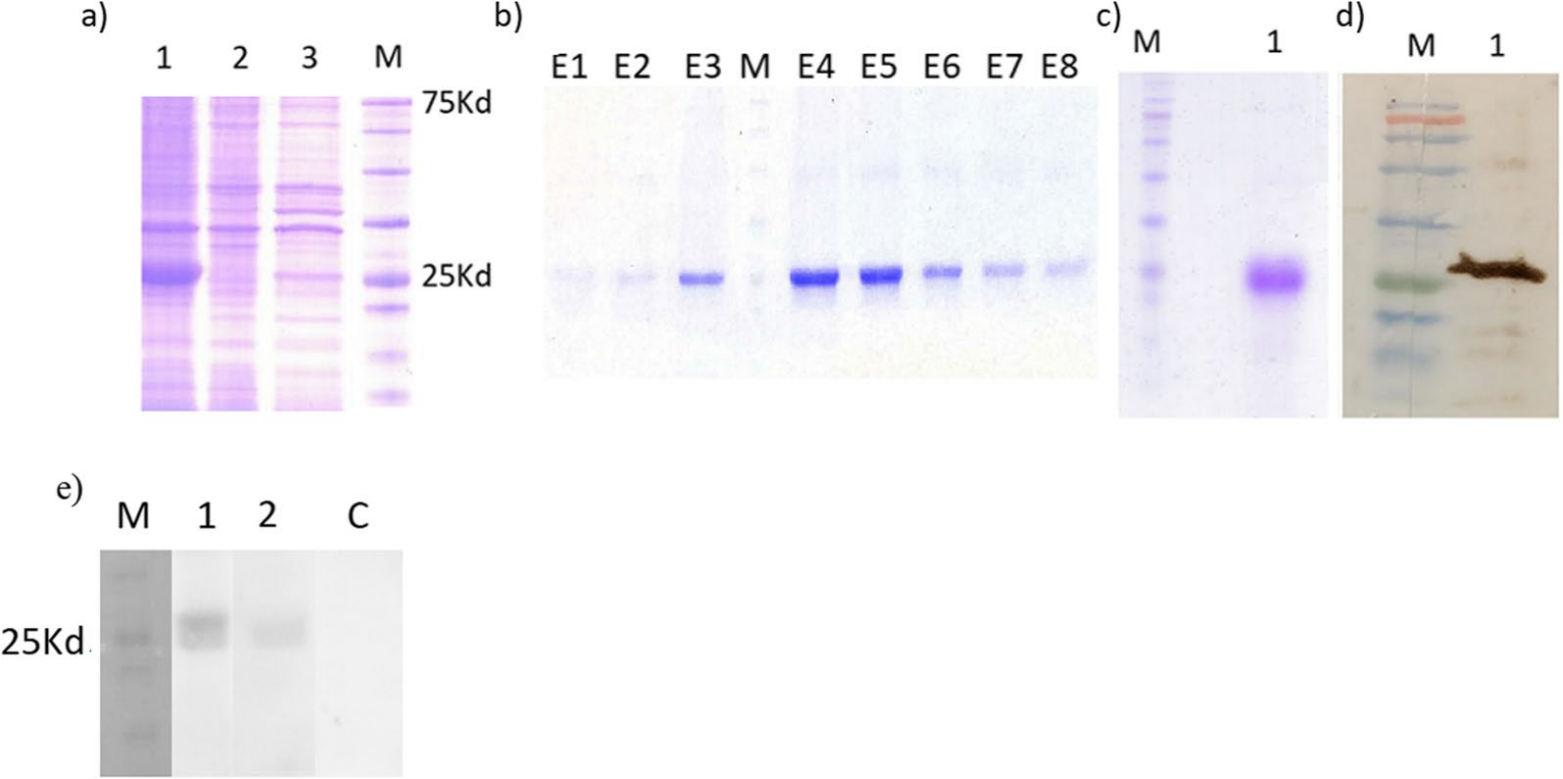
**a** Expression of RBD in BL21(DE3) in the presence of 0.1 mM IPTG for 3 h (lane 1), uninduced BL21(DE3) (lane 2), BL21(DE3) control in the absence of IPTG (lane 3), marker (M). **b** Fractions of rRBD purification using Ni column, **c** Purity assessment of RBD (10 µg) by SDS-PAGE, **d** Western blot analysis of rRBD using anti-His tag antibody, and **e** two SARS-CoV-2 vaccinated human serums; positive serums (lane1 and 2), negative serum (C)

The BL21(DE3) was transformed with the rRBD construct. The optimum expression level of rRBD was obtained in the presence of 1 mM IPTG after 3 h induction at 37 °C. The rRBD protein as inclusion bodies was expressed in 50 ml LB medium (Fig. 1a). The protein extraction from inclusion bodies was performed using denaturing buffer, including high concentrations (8 M) of urea. SDS-PAGE electrophoresis exhibited that the molecular weight of the purified rRBD protein was about 27 kDa with high purity (Fig. 1b, c). The BCA method revealed a 250 µg/ml concentration of purified protein. The western blot analysis using anti-His antibody indicated the presence of a distinct band at 27 kDa, which confirmed the identity of the purified protein as the rRBD (Fig. 1d). In addition, the characterization of the rRBD protein was carried out using western blot analysis with both a positive and negative human serum. The western blot results showed that rRBD was recognized by SARS-CoV-2 immunized human serum. No band was observed with negative human serum, indicating the specificity of the reaction (Fig. 1e). ELISA assay confirmed the binding of ACE2 to rRBD, as shown in Additional file 1. In addition, in all mAbs neutralization assays, ACE2 competes with antibodies for binding to rRBD.

### Immunization of mice, screening and selection strategy of anti-rRBD hybridoma clones

**Fig. 2:**
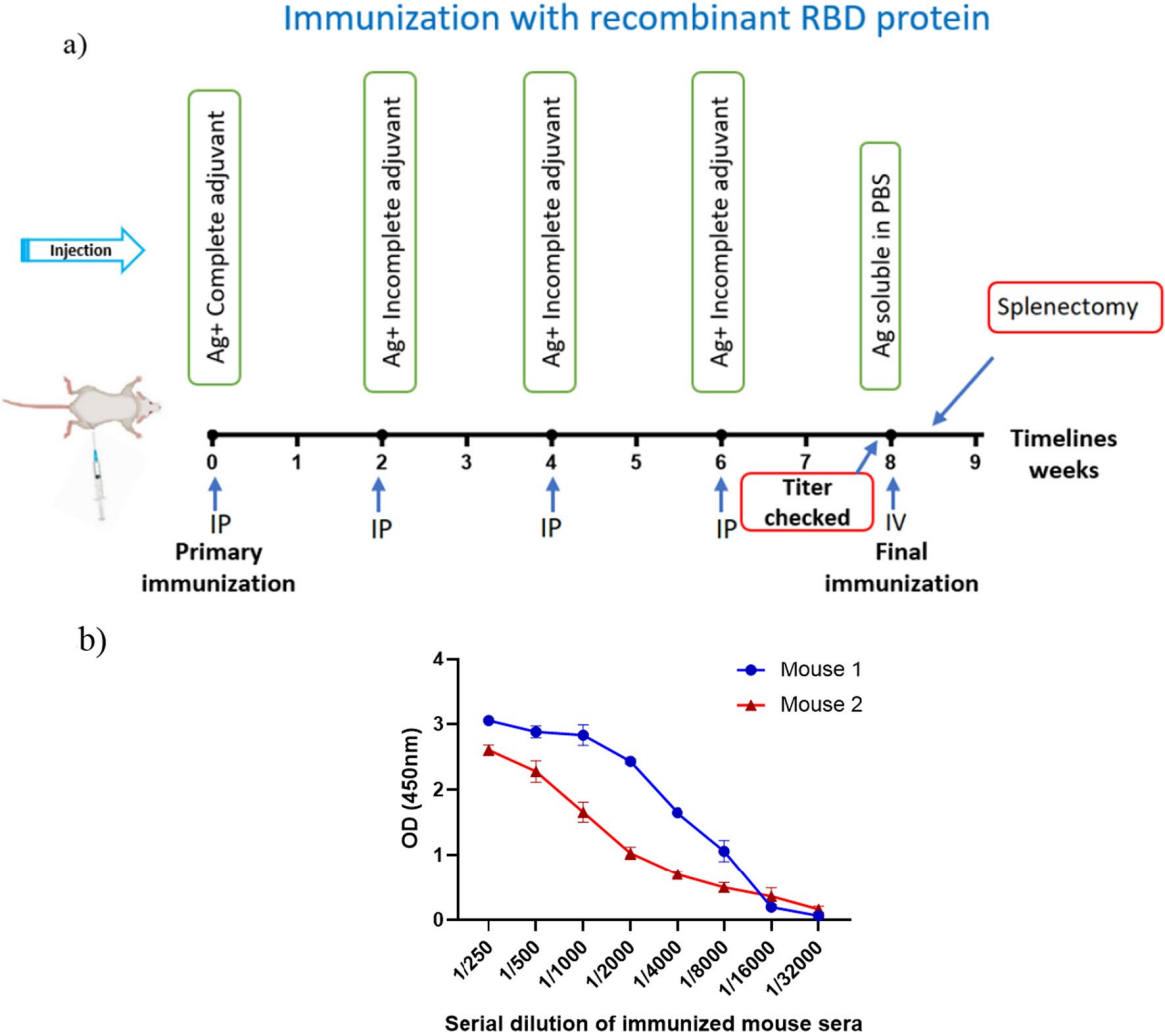
Balb/c immunization strategy and anti-rRBD-specific IgG titration curves. **a** Immunization timeline. Balb/c mice were immunized with rRBD protein. Immunogens were injected via IP and IV. **b** Titration of anti-rRBD-specific antibodies in mouse sera using indirect ELISA. The hyperimmunized mouse was selected for final immunization, followed by splenectomy for hybridoma generation

Following mouse immunization using rRBD (Fig. 2a), the anti-rRBD antibody was detected in mouse sera by ELISA, and the mouse with a higher titer of specific antibodies was selected for the final immunization (Fig. 2b).

Several hybridoma colonies were observed within 7 days after being cultured in a HAT medium. Afterward, all wells containing hybridoma colonies were screened by indirect ELISA, and 5 clones producing anti-rRBD were selected for serial dilution. Therefore, using these screening strategies, 5 hybridomas (2B4-G11, 1D9-D1, 1G1-G9, 2E7-D2, and 2B4-G8) were successfully obtained that producing anti-rRBD mAb and expanded in serum-medium for anti-rRBD production.

### Purity assessment and characterization of anti-rRBD mAbs

#### SDS-PAGE

**Fig. 3:**
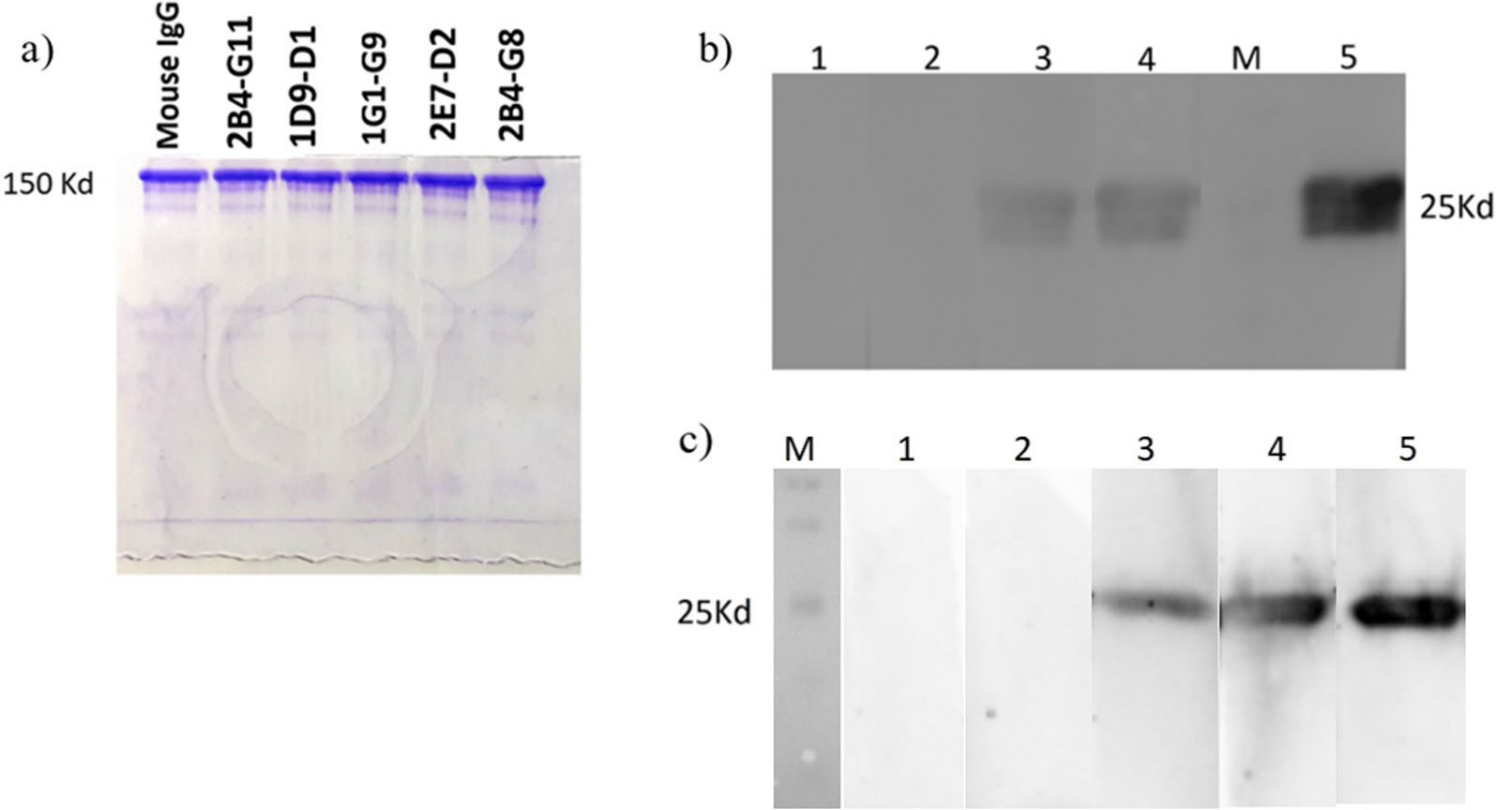
**a** SDS-PAGE analysis of anti-SARS-CoV-2-RBD mAbs. Mouse IgG, 2B4-G11, 1D9-D1, 1G1-G9, 2E7-D2, 2B4-G8; the purified mAbs of anti-SARS-CoV-2-RBD. **b** Detection of rRBD protein using anti-rRBD mAbs by non-reducing western blot. Each lane was incubated with mAb as follows: Lane 1: 2B4-G11, Lane 2: 1D9-D1, Lane 3: 1G1-G9, Lane 4: 2E7-D2, Lane 5: 2B4-G8. **c** Detection of rRBD protein using anti-rRBD mAbs by reducing western blot: Lane 1: 2B4-G11, Lane 2: 1D9-D1, Lane 3: 1G1-G9, Lane 4: 2E7-D2, Lane 5: 2B4-G8

The electrophoretic pattern of purified mAbs is shown in Fig. 3a. The results indicated a 150 kDa band of mouse IgG and a high purity of mAbs.

#### Reactivity assays

##### Western blot

The rRBD was electrophoresed under reducing and non-reducing western blots. After protein transfer to the PVDF membrane, the membrane was incubated with purified mAbs. Three mAbs (1G1-G9, 2E7-D2, and 2B4-G8) were found to be capable of detecting reducing and non-reducing rRBD, demonstrating that these antibodies recognize linear epitopes, while no reactivity was observed when 2B4-G11 and 1D9-D1 were applied, indicating the recognition of conformational epitopes by these antibodies (Fig. 3b and c). Based on the western blot analysis, 2B4-G8 showed a stronger signal, indicating a higher reactivity than the other mAbs.

##### ELISA

**Fig. 4:**
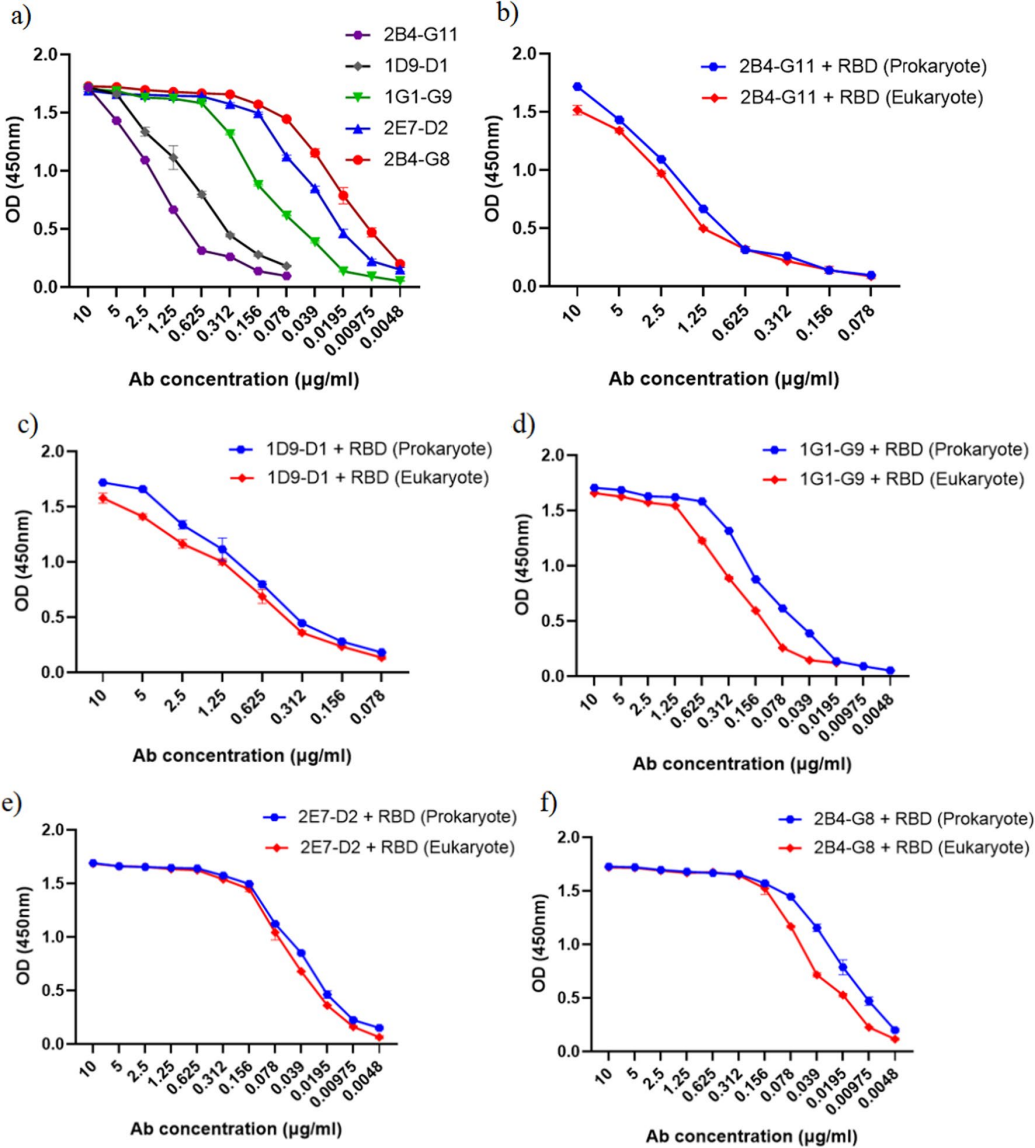
Reactivity assessment of purified anti-rRBD mAbs. The purified mAbs (2B4-G11, 1D9-D1, 1G1-G9, 2E7-D2, and 2B4-G8) were serially diluted against the rRBD and eukaryotic RBD by the ELISA assay. **a** Reactivity comparison of mAbs using rRBD coated plates. **b, c, d, e, f** Reactivity comparison of each mAb using rRBD and eukaryotic RBD

All purified mAbs detected prokaryotic (Fig. 4a) and eukaryotic RBD in ELISA assay (Fig. 4b–f) with different reactivity. The reactivity profile of all mAbs against rRBD is shown in Fig. 4a. 2E7-D2, 2B4-G8, 1G1-G9, 1D9-D1, and 2B4-G11 mAbs demonstrated similar reactivity trends against the eukaryotic RBD and the prokaryotic rRBD. The ELISA results showed higher reactivity of 2E7-D2 and 2B4-G8 mAbs compared to other mAbs, reaching a plateau at 0.156 µg/ml concentrations.

##### Affinity assay

**Fig. 5:**
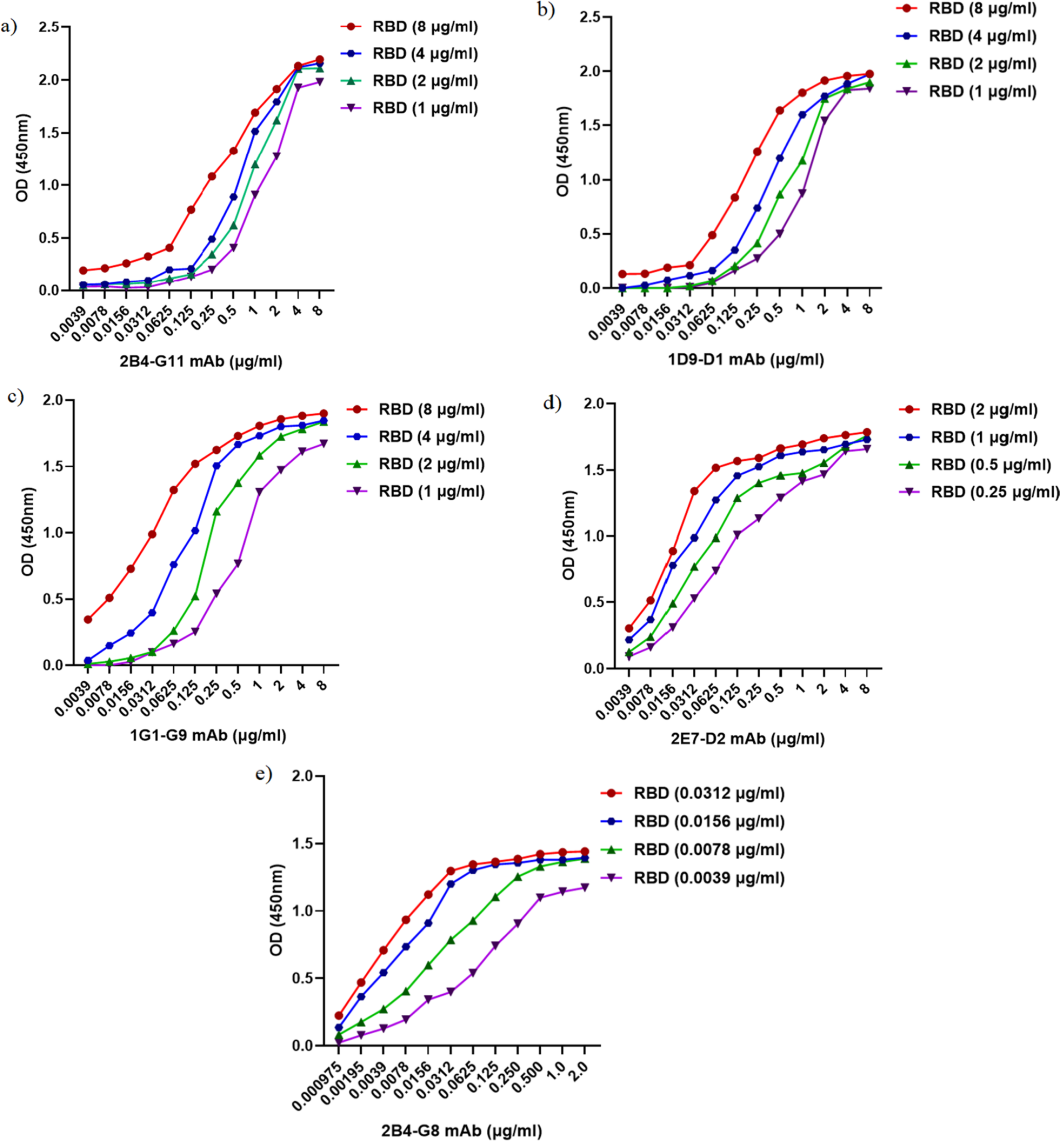
Determination of affinity constant of mAbs (2B4-G11, 1D9-D1, 1G1-G9, 2E7-D2, and 2B4-G8) (K_aff_) by ELISA. **a, b, c, d, e** Different concentrations of mAbs were tested against serial dilutions of rRBD protein and K_aff_ was calculated

The affinity constant of mAbs was determined using ELISA. The affinity indicates the binding strength between the mAbs and the target protein. A higher affinity value signifies a more robust interaction, indicating a higher potential for effectively targeting the rRBD protein. Due to the high reactivity of the 2B4-G8 antibody compared to other mAbs and the fact that the OD50 (half maximum optical density) is necessary for the affinity measurement, lower concentrations of this antibody were applied in the affinity determination test. Moreover, the affinity of these mAbs (2B4-G11, 1D9-D1, 1G1-G9, 2E7-D2, and 2B4-G8) were determined 4.4 × 10^8^, 6.4 × 10^8^, 1.87 × 10^9^, 1.139 × 10^10^ and 2.43 × 10^10^ M^−1^ respectively (Fig. 5 and Table 1).

**Table 1:** Affinity of mAbs determined by ELISA

mAb Name	[Ag] (μg/ml)	OD-50*	Average K_aff_ (M^-1^)
2B4-G11	8.0 4.0 2.0 1.0	1.0965 1.078 1.0545 0.99	4.4×10^8^
1D9-D1	8.0 4.0 2.0 1.0	0.988 0.987 0.9495 0.92	6.4×10^8^
1G1-G9	8.0 4.0 2.0 1.0	0.9495 0.923 0.9195 0.8355	1.87×10^9^
2E7-D2	2.0 1.0 0.5 0.25	0.894 0.866 0.8805 0.8295	1.139×10^10^
2B4-G8	0.0312 0.0156 0.0078 0.0039	0.721 0.697 0.694 0.586	2.43×10^10^

^*^OD-50 represents the half maximum optical density obtained for a given concentration of rRBD ([Ag]) and the corresponding mAb ([Ab]). The affinity constant (*K*_*aff*_) for each selected concentration of Ag and Ab was calculated using the formula described in the Methods

### Neutralizing activity of mAbs

**Fig. 6:**
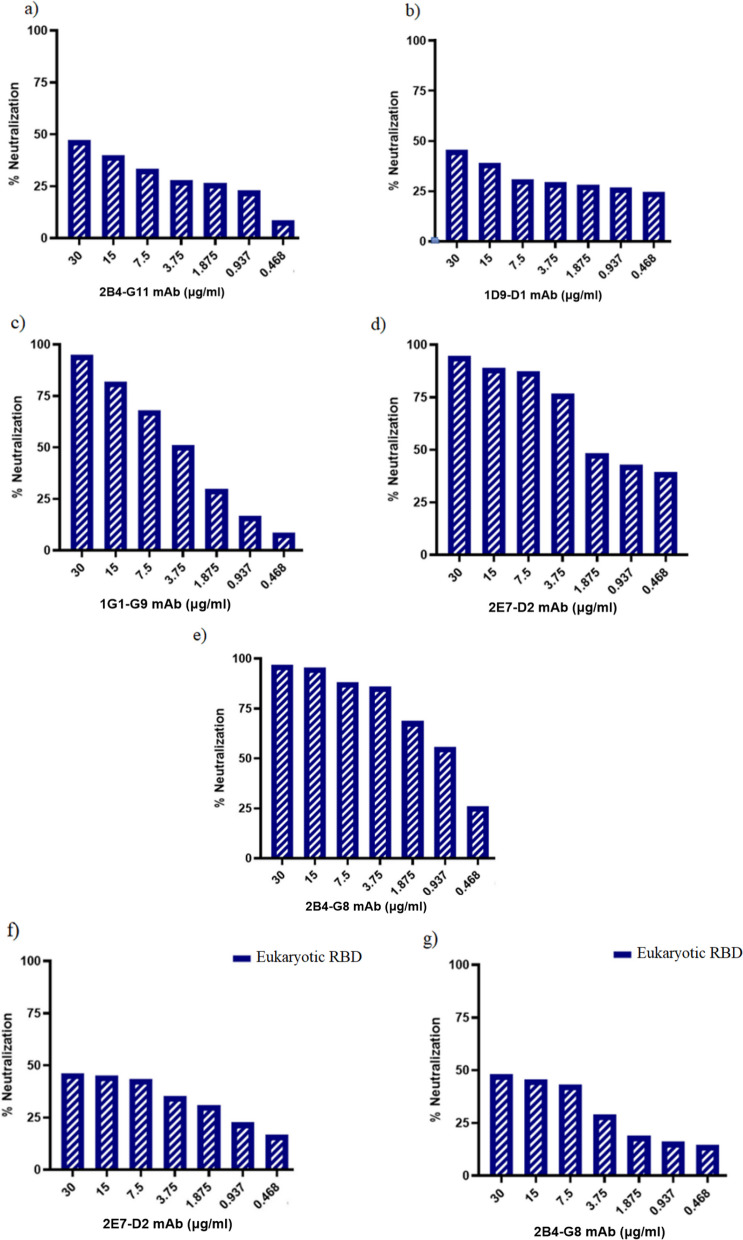
The neutralization percent of anti-rRBD mAbs. The purified mAbs (2B4-G11, 1D9-D1, 1G1-G9, 2E7-D2, and 2B4-G8). Measurement of mAbs neutralization activity using rRBD **a, b, c, d, e** and eukaryotic RBD **f, g** blocking ELISA assay

The neutralizing activity of 5 mAbs were further determined by blocking ELISA assay. The inhibitory effect of mAbs anti-rRBD on RBD/ACE2 binding was evaluated using the rRBD/ACE2 blocking ELISA assay. According to the results, all mAbs inhibit the interaction between rRBD and ACE2 (Fig. 6a–e). At the maximum antibody concentration (30 µg/ml), three antibodies (2E7-D2, 2B4-G8, and 1G1-G9) showed approximately 100% neutralization, while the other two antibodies exhibited 50% neutralization activity at the same concentration. The neutralization was dependent on the antibody concentration. As seen in Fig. 6, the neutralization percentage reduced with decreasing antibody concentration. Similarly, the neutralizing activity of mAbs were evaluated using eukaryotic RBD (Fig. 6f–g). 2E7-D2 and 2B4-G8 mAbs exhibited relatively high neutralizing activity, while 2B4-G11, 1D9-D1, and 1G1-G9 could not neutralize eukaryotic RBD even at high antibody concentrations. Characteristics of all mAbs were summarized in Table 2.

**Table 2:** Characterization of anti-rRBD monoclonal antibodies

mAb (clone)	Reactivity assessment with prokaryotic rRBD by ELISA	Reactivity assessment with eukaryotic RBD by ELISA	Neutralization with prokaryotic rRBD by blocking ELISA	Neutralization with blocking eukaryotic RBD by ELISA	Reactivity assessment with prokaryotic rRBD by western blot
2B4-G11	2 +	2 +	2 +	Neg	Neg
1D9-D1	2 +	2 +	2 +	Neg	Neg
1G1-G9	3 +	3 +	4 +	Neg	Pos (2 +)
2E7-D2	4 +	4 +	4 +	2 +	Pos (2 +)
2B4-G8	4 +	4 +	4 +	2 +	Pos (4 +)

^*^The reactivity was presented as (*Neg* Negative reactivity, 1 + : Weak reactivity, 2 + : Moderate reactivity, 3 + : Strong reactivity, and 4 + : Very strong reactivity)

## Discussion

The COVID-19 pandemic and the emergence of new SARS-CoV-2 variants have required the rapid development of beneficial therapies [20]. Since, at the time of the study's design in 2021, the dominant variant of SARS-CoV-2 was delta [21], delta-rRBD was selected to develop potent NmAbs. Due to the sequence [22] and structural [23] similarities between the RBD domain of delta and omicron variants, the produced mAbs targeting the RBD domain of the delta variant may have the potential to recognize and neutralize the omicron variants [22, 23]. The crystal 3D structure of the omicron and delta RBD is reported to exhibit a similar conformation. Both flow cytometry and surface plasmon resonance assays showed that the binding capacity and affinities of the omicron and delta RBDs to hACE2 have not significantly altered [23].

We successfully produced and purified rRBD protein in this study using an E. coli expression system. There are differences between eukaryotic and prokaryotic expression systems. When the protein is expressed in mammalian cells, it can pose challenges to acquiring an adequate quantity of the antigen required for animal injection [24]. Some studies have reported the production of RBD protein using mammalian cells or insect cells [25, 26]. However, most of them are time-consuming and costly. Prokaryotic expression systems have several advantages, including rapid and low-cost production, higher yield, and easy ways to large-scale production, which may be useful in developing countries with limited resources [27, 28]. Although it seems that the folding and structure of the eukaryotic RBD proteins are more similar to the virus's proteins, both prokaryotic and eukaryotic RBDs elicit virus-neutralizing cross-specific IgG antibodies [29]. After rRBD production and purification, Coomassie-stained SDS/PAGE gel analysis (10 µg/well) revealed no detectable contamination, representing a single protein band with the expected size (27 kDa) (Fig. 1c). This level of purity is ideal for producing antibodies [30]. Based on the reasons given, prokaryotic rRBD was used to produce mAbs with neutralizing specificity.

One of the therapeutic or prophylactic treatments against SARS-CoV-2 is using neutralizing antibodies, which have advantages such as specificity (to target virus or antigen), potency (effective at low concentrations), and durability (long-lasting in the body) [31, 32]. NmAbs against the RBD, such as REGN-COV2 and LY-CoV555, can inhibit viral replication and spread by preventing or reducing the binding and entry of the virus into cells [33, 34].

Several methods have been used to generate antibodies against SARS-CoV-2, including the phage display, B cell selection from COVID-19 patients using techniques such as single B cell PCR and single B cell FACS sorting. Additionally, mAbs are produced from transgenic mice and the hybridoma technique [20, 35–38].

Hybridoma technology provides limitless production of cost-effective, highly pure, specific, and homogenized monoclonal antibodies with a high affinity to the epitope of targeted antigens [39, 40]. There are some limitations or disadvantages in the mentioned techniques. For example, in phage display, antigen binding loss and poor yield are common problems [41]. Thus, this study focused on hybridoma generation, selection, screening, and purification procedure to develop murine-neutralizing mAbs using rRBD protein, which can be potentially used to treat and diagnose the SARS-CoV-2 antigen.

Five distinct hybridoma clones (2B4-G11, 1D9-D1, 1G1-G9, 2E7-D2, and 2B4-G8) were successfully generated and characterized. These clones were found to secrete mAbs that specifically recognized the rRBD in ELISA. All mAbs have shown high reactivity with different concentrations of rRBD in ELISA. In addition, the higher reactivity of 2E7-D2 and 2B4-G8 may stem from its relatively higher affinity. Some studies have characterized high-affinity antibodies, ranging from 1.38 to 21.29 nM, comparable to 2E7-D2 and 2B4-G8 mAbs [38, 42].

Our findings represented that the 2B4-G11 and 1D9-D1 mAbs, unlike other clones, could not detect the rRBD protein in western blot, indicating that these antibodies recognize conformational epitopes on RBD. Conversely, 1G1-G9, 2E7-D2, and 2B4-G8 mAbs bound to rRBD in western blot after denaturing of RBD using SDS and reducing with 2ME, indicating the recognition of linear epitopes [43] by these antibodies.

The immunoreactivity of mAbs, including 2B4-G11, 1D9-D1, 1G1-G9, 2E7-D2, and 2B4-G8, were also examined using eukaryotic RBD, which showed high reactivity patterns with eukaryotic RBD in ELISA. Considering that the high immunoreactivity of 2E7-D2 and 2B4-G8 mAbs with both RBDs in ELISA and also recognition of RBD linear epitopes when evaluated by western blot, it can be concluded that these clones recognize common epitopes in rRBD and eukaryotic RBD.

We introduce NmAbs against SARS-CoV-2 with linear epitope recognition specificity. In line with our results, a recent study reported mAbs with strong neutralizing activities directed to linear epitopes [44]. However, there are limited reports on the serological reactivity to "linear" immunodominant sites on the RBD [45]. mAbs that recognize the linear epitopes on the RBD can be highly valuable due to their advantageous role in developing epitope-based vaccines [45, 46]. Based on epitope mapping, studies have shown that most NmAbs are largely directed to conformational epitopes in RBM [45, 47–49]. Of note, 2B4-G11, 1D9-D1, and 1G1-G9 mAbs showed no neutralization activity with eukaryotic RBD. This may be due to the difference in the structure and folding of recombinant RBDs produced in prokaryotic systems compared to eukaryotic systems. Eukaryotic RBDs are probably different from prokaryotic RBDs in terms of folding due to the lack of glycosylation and disulfide bond formation in the E. coli expression system [50]. Numerous studies revealed disulfide bonds as structural elements in determining the final three-dimensional structure of proteins [51]. Specific regions of proteins, such as alpha helix or beta sheet, can be stabilized by disulfide bonds. Disulfide bonds hold the structure in place and maintain the overall three-dimensional structure of the protein [51, 52].

2E7-D2 and 2B4-G8 mAbs at 30 μg/ml concentration have shown different neutralizing reactivity with rRBD (100% inhibition rate) and eukaryotic RBD (50% inhibition rate). A published study has shown that antibodies with 50–85% neutralizing activity in ELISA can neutralize the virus [53]. Another study reported that the potent neutralizing mAbs achieved less than 100% inhibition rate in the ELISA assay [54]. According to the results, all mAbs efficiently inhibit the interaction between rRBD and ACE2 (Fig. 6a–e), while the neutralizing rate was less with eukaryotic RBD. The neutralizing activity of antibodies depends on structure, folding, and post-translational modifications of RBD. It should be noted that these parameters can be varied between prokaryotic and eukaryotic expression systems [29].

This study was focused only on the delta strain, limiting the generalizability of the findings to other strains. To address this, future research should include pseudovirus experiments to verify the cross-protection of the mAbs against different SARS-CoV-2 variants. Moreover, due to limited access to Biosafety Level 3 (BSL-3), we could not perform the Virus Neutralizing Test (VNT) in this study.

## Conclusion

In the current study, we generated and characterized new RBD-specific mAbs using the hybridoma technique that recognized linear and conformational epitopes in RBD with neutralization potency. Our mAbs are novel candidates for diagnosing and treating SARS-CoV-2.

### Supplementary Information

**Additional file 1 Fig. S1 **Reactivity assessment of rRBD binding to human ACE2. rRBD was detected by ACE2-HRP in ELISA. The experiment was performed in duplicates and the mean value is given.

## Data Availability

Not applicable.

## Abbreviations

ACE2: Angiotensin-converting enzyme-2
BCA: Bicinchoninic acid
BSA: Bovine serum albumin
DAB: 3,3′-Diaminobenzidine
ECL: Enhanced chemiluminescence
HAT: Hypoxanthine-aminopterin-thymidine medium
HRP: Horseradish peroxidase
IPTG: Isopropyl-β- D-Thio-Galactopyranoside
IP: Intraperitoneal
mAb: Monoclonal antibody
NmAbs: Neutralizing monoclonal antibodies
nAbs: Neutralizing antibodies
NTD: N-terminal domain
OD: Optical density
PVDF: Polyvinylidene difluoride
PEG: Polyethylene glycol
RBD: Receptor-binding domain
rRBD: Recombinant RBD
SARS-CoV-2: Severe acute respiratory syndrome coronavirus 2
