# Supplementary material for: Novel neutralizing SARS-CoV-2-specific mAbs offer detection of RBD linear epitopes
Source: Virol J. 2024 Feb 6;21:37. doi: 10.1186/s12985-024-02304-2 (PMC10845636; doi:10.1186/s12985-024-02304-2)
Supplement: Supplementary file 1 — Additional file 1 Fig. S1 Reactivity assessment of rRBD binding to human ACE2. rRBD was detected by ACE2-HRP in ELISA. The experiment was performed in duplicates and the mean value is given. [file 12985_2024_2304_MOESM1_ESM.docx]

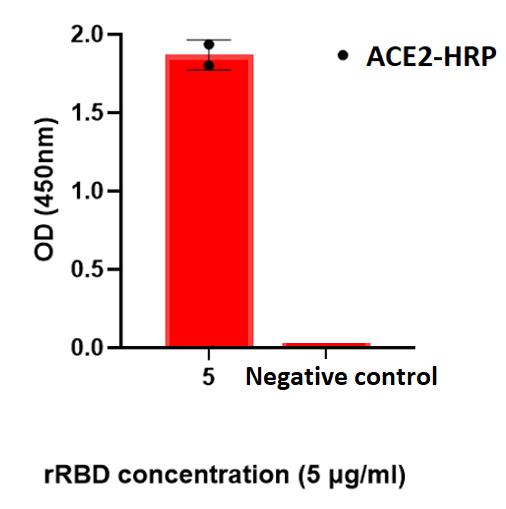


Supplementary 1. Reactivity assessment of rRBD binding to human ACE2. rRBD was detected by ACE2-HRP in ELISA. The experiment was performed in duplicates and the mean value is given.
